# Pro-inflammatory Monocyte Phenotype During Acute Progression of Cerebral Small Vessel Disease

**DOI:** 10.3389/fcvm.2021.639361

**Published:** 2021-05-13

**Authors:** Marlies P. Noz, Annemieke ter Telgte, Kim Wiegertjes, Anil M. Tuladhar, Charlotte Kaffa, Simone Kersten, Siroon Bekkering, Charlotte D. C. C. van der Heijden, Alexander Hoischen, Leo A. B. Joosten, Mihai G. Netea, Marco Duering, Frank-Erik de Leeuw, Niels P. Riksen

**Affiliations:** ^1^Department of Internal Medicine, Radboud Institute for Molecular Life Science, Radboud University Medical Center, Nijmegen, Netherlands; ^2^Department of Neurology, Donders Institute for Brain, Cognition and Behaviour, Radboud University Medical Center, Nijmegen, Netherlands; ^3^Center for Molecular and Biomolecular Informatics, Radboud Institute for Molecular Life Sciences, Radboud University Medical Center, Nijmegen, Netherlands; ^4^Department of Human Genetics, Radboud Institute for Molecular Life Sciences, Radboud University Medical Center, Nijmegen, Netherlands; ^5^Department of Medical Genetics, Iuliu Hatieganu University of Medicine and Pharmacy, Cluj-Napoca, Romania; ^6^Department for Genomics and Immunoregulation, Life and Medical Sciences Institute, University of Bonn, Bonn, Germany; ^7^Institute for Stroke and Dementia Research, University Hospital of Munich, Munich, Germany; ^8^Munich Cluster for Systems Neurology, Munich, Germany

**Keywords:** cerebral small vessel disease, magnetic resonance imaging, innate immunity, monocyte, inflammation

## Abstract

**Background:** The etiology of cerebral small vessel disease (SVD) remains elusive, though evidence is accumulating that inflammation contributes to its pathophysiology. We recently showed retrospectively that pro-inflammatory monocytes are associated with the long-term progression of white matter hyperintensities (WMHs). In this prospective high-frequency imaging study, we hypothesize that the incidence of SVD progression coincides with a pro-inflammatory monocyte phenotype.

**Methods:** Individuals with SVD underwent monthly magnetic resonance imaging (MRI) for 10 consecutive months to detect SVD progression, defined as acute diffusion-weighted imaging-positive (DWI+) lesions, incident microbleeds, incident lacunes, and WMH progression. Circulating inflammatory markers were measured, cytokine production capacity of monocytes was assessed after *ex vivo* stimulation, and RNA sequencing was performed on isolated monocytes in a subset of participants.

**Results:** 13 out of 35 individuals developed SVD progression (70 ± 6 years, 54% men) based on incident lesions (*n* = 7) and/or upper quartile WMH progression (*n* = 9). Circulating E-selectin concentration (*p* < 0.05) and the cytokine production capacity of interleukin (IL)-1β and IL-6 (*p* < 0.01) were higher in individuals with SVD progression. Moreover, RNA sequencing revealed a pro-inflammatory monocyte signature including genes involved in myelination, blood–brain barrier, and endothelial–leukocyte interaction.

**Conclusions:** Circulating monocytes of individuals with progressive SVD have an inflammatory phenotype, characterized by an increased cytokine production capacity and a pro-inflammatory transcriptional signature.

## Introduction

Cerebral small vessel disease (SVD) is a common condition in elderly individuals and is the most important vascular contributor to dementia, lacunar infarcts, Parkinsonism, and ultimately loss of independence (1–3). SVD affects the structure and function of the smallest cerebral blood vessels, including the arterioles, capillaries, and venules of the brain, resulting in brain parenchymal tissue changes (4). Tissue alterations thought to arise from SVD are mainly detected with magnetic resonance imaging (MRI) and include diffusion-weighted imaging-positive (DWI+) lesions suggestive of acute (micro)infarcts, microbleeds, lacunes, and white matter hyperintensities (WMHs), among others (5). Imaging of small cerebral vessels is difficult, and the inability to visualize the initiation of arteriolar pathology has arguably contributed to the fact that the etiology of SVD has remained elusive. In the past few years, evidence is compiling on the role of inflammation in SVD pathophysiology.

Inflammation is increasingly acknowledged as a risk factor for SVD (6). Meta-analyses reported elevated circulating markers of inflammation, e.g. interleukin 6 (IL-6) and C-reactive protein (CRP), and markers of endothelial dysfunction, e.g. E-selectin, in individuals with SVD (7–9). In addition, longitudinal studies demonstrated that systemic inflammatory markers at baseline predicted subsequent SVD severity (10, 11). Inflammation and endothelial dysfunction contribute to chronic disruption of the blood–brain barrier, which is thought to aggravate SVD (12, 13). Blood–brain barrier disruption enhances leakage of signaling mediators and facilitates the communication between circulating and tissue-resident immune cells.

Circulating innate immune cells, capable to produce circulating inflammatory markers, might have a significant influence on the development of SVD. We recently showed in a retrospective analysis that a pro-inflammatory phenotype of circulating monocytes was associated with the progression and severity of SVD (14). In detail, the cytokine production capacity of monocytes strongly correlated with the progression of WMH over a 9-year course (14). The systemic inflammation marker IL-6 (high-sensitive (hs)IL-6) and the pro-inflammatory (CD14^++^CD16^+^) intermediate monocyte subset correlated with the WMH progression rate in individuals with SVD. However, the retrospective approach of this study precludes the assessment of a direct relation between pro-inflammatory monocytes and inflammatory markers with the development of SVD.

In this study, we aimed to further elucidate the role of innate immune activation in SVD by investigating whether the occurrence of SVD progression is linked to the circulating monocyte phenotype. Therefore, high-frequency serial imaging with MRI was performed monthly for 10 consecutive months to detect multiple imaging markers of SVD, including the incidence of acute (micro)infarcts (defined as DWI+ lesions), microbleeds, lacunes, and WMH. In addition, we aimed to provide a deeper understanding of the inflammatory phenotype of circulating monocytes by performing RNA sequencing of monocytes from individuals with SVD progression compared to individuals without signs of SVD progression.

## Materials and Methods

The data that support the findings of this study are available from the corresponding author upon reasonable request.

### Study Design and Participants

Individuals were enrolled in the RUN DMC–InTENse study (Radboud University Nijmegen Diffusion tensor and Magnetic resonance imaging Cohort–Investigating The origin and EvolutioN of cerebral small vessel disease), a longitudinal observational study, comprising a pre-visit, 10 MRI visits over a period of 10 months, and a follow-up visit 1 year after the pre-visit (15).

The major inclusion criterion was progression of WMH between 2006 and 2015, as demonstrated on MRI collected within the previous RUN DMC study (16). To rule out causes of cerebral ischemia other than SVD, exclusion criteria for the RUN DMC–InTENse study were the presence of large artery disease defined as carotid artery stenosis (>50% assessed by carotid ultrasound), atrial fibrillation or the use of oral anticoagulants, previous cortical ischemic stroke or transient ischemic attack, intracranial hemorrhage (other than a microbleed), and large artery vasculitis. Participants with dementia or Parkinson's disease [according to Diagnostic and Statistical Manual of Mental Disorders (DSM)-IV criteria] were also excluded from participation. Furthermore, in the current study, we excluded participants with autoimmune/inflammatory diseases including diabetes mellitus, or daily immunomodulatory drug use, because these conditions interfere with the immune response. All individuals gave written informed consent. The study protocol was approved by the Institutional Review Board Arnhem/Nijmegen, Netherlands (NL53939.091.15).

### MRI Acquisition and Image Processing

A detailed description of the MRI protocol and image analysis was recently published (17, 18). Briefly, individuals were scanned using a single 3 T MRI system (MAGNETOM Prisma, Siemens Healthineers, Erlangen, Germany) and a 32-channel head coil. To detect DWI+ lesions, we obtained multi-shell DWI scans (90 diffusion-weighted directions including 30 × b = 1,000, and 60 × b = 3,000 s/mm^2^, 10 b = 0 images, and voxel size 1.7 × 1.7 × 1.7 mm). Trace images were created for the b = 1,000 and b = 3,000 shells, and a mean diffusivity map was calculated for the b = 1,000 shell. We acquired 3D multi-echo fast low angle shot images (6 echoes, voxel size 0.8 × 0.8 × 2.0 mm), which were used to create susceptibility-weighted images to identify cerebral microbleeds. Finally, to assess WMH and lacunes, we acquired 3D fluid-attenuated inversion recovery images (voxel size 0.85 × 0.85 × 0.85 mm) and 3D T1-weighted images (voxel size 0.85 × 0.85 × 0.85 mm).

#### Incident Small Vessel Disease Lesion Detection

Detection of SVD imaging markers was done according to previously established criteria (5). Briefly, DWI+ lesions were manually detected and defined as hyperintense lesions on diffusion-weighted trace images (both b = 1,000 and b = 3,000), accompanied by a hypointense or isointense signal on the mean diffusivity map. Microbleeds were detected semiautomatically as hypointense lesions on the FLASH images. Incident lacunes (including cavities < 3 mm) were manually detected using difference FLAIR and T1-weighted images, generated by subtracting registered baseline FLAIR and T1 scans from the last available follow-up scans. WMHs were automatically segmented based on a deep-learning algorithm utilizing FLAIR and registered T1-weighted images as input images. For each case, the WMH volumes extracted from the monthly scans were corrected for white matter volume extracted from the corresponding visit. Next, in a simple linear regression model on WMH volume over time, we calculated the predicted individual WMH volume at each time point and progression of WMH (i.e., the slope of the regression model).

Individuals with any incident lesion during the study period (DWI+ lesion, microbleed, or lacune) and individuals belonging to the first quartile of WMH progression were classified as participants with SVD progression.

### Cardiovascular Risk Assessment

At study inclusion, medical history, medication use, and cardiovascular risk assessment, including measurement of the blood pressure three times [according to American Heart Association (AHA) guidelines (19)], smoking status, body mass index (BMI), and capillary non-fasting glucose was obtained.

### Blood Sampling

Parallel to the third MRI appointment [median of 16 [12–23] weeks after pre-visit], overnight fasted blood was obtained in EDTA vacutainers between August 2016 and February 2017. Plasma and serum were stored in −80°C. Total cholesterol (Tchol), high-density lipoprotein cholesterol (HDL-C), and triglycerides (TGs) were measured using standardized methods. Low-density lipoprotein cholesterol (LDL-C) was calculated with the Friedewald formula. Total blood counts were measured with an automated cell counter using Sysmex-XN 450 hematology analyzer (Sysmex, Hamburg, Germany).

### Monocyte Isolation and Stimulation

Monocytes were isolated using Ficoll-Paque density gradient centrifugation (GE Healthcare, Chicago, USA) followed by magnetic activated cell sorting using the Pan Monocyte Isolation kit according to manufacturer's instructions (Miltenyi Biotec, Bergisch Gladbach, Germany). Cell purity of the monocyte fraction [median 92% (88–95%) monocytes-of-leukocytes] was evaluated with flow cytometry. Monocytes were concentrated in RPMI 1640 Dutch-modified culture medium (Life Technologies/Invitrogen, Waltham, USA) supplemented with 2 mmol/L glutamine (Invitrogen), 10 mg/ml gentamicin (Centrafarm, Etten-Leur, Netherlands), and 1 mmol/L pyruvate (Invitrogen). 1 × 10^5^ monocytes were seeded on flat-bottom 96-well plates (Corning, New York, USA) and stimulated in triplicate for 24 h with RPMI (control), toll-like receptor 4 agonist lipopolysaccharide (LPS) from *E. coli* (10 ng/ml, serotype 055:B5; Sigma-Aldrich, St. Louis, USA), and toll-like receptor 2 agonist Pam3CysK4 (P3C) (10 μg/ml, L2000; EMC Microcollections, Tübingen, Germany). Supernatants were collected after plate centrifugation and stored in −80°C freezer until cytokine assessment.

### Cytokine Assessment

Cytokine and chemokine concentrations were measured in plasma and in supernatants after stimulation with enzyme-linked immunosorbent assay (Supplementary Table 1).

### Flow Cytometry

Monocyte subpopulations, consisting of CD14^++^CD16^−^ classical monocytes, CD14^++^CD16^+^ intermediate monocytes, and CD14^+^CD16^+^ non-classical monocytes, were identified with the FC500 flow cytometry (Beckman Coulter, Brea, USA) using the lysis-no-wash strategy (BD Pharm Lyse lysing buffer, Becton Dickinson) with 100 μl fresh EDTA blood. Cells were stained by monoclonal antibodies (CD16 FITC Leu11a, Becton&Dickinson; CD14 ECD RM052, Beckman-Coulter; CD45 PC5 J.33, Beckman-Coulter) and subsequently analyzed with Kaluza software version 1.5a (Beckman Coulter).

The full gating strategy is displayed in Supplementary Figure 1. In short, monocytes were gated in SSC/CD45^+^ plot, identifying monocytes as CD45^+^ cells with monocyte scatter properties. Exclusion of lymphocytes and natural killer cells was performed by excluding CD14/CD16 negative cells. Percentages of monocyte subsets (CD14^++^CD16^−^, CD14^++^CD16^+^, and CD14^+^CD16^+^) were identified in the CD14/CD16 plot. For determination of the gates setting, the fluorescence minus one method was applied. Identification of monocyte subsets follows current international recommendations (20, 21).

### RNA Isolation and Quantseq 3′ mRNA Sequencing

RNA sequencing was completed for four participants with SVD progression [composing of DWI+ lesions (*n* = 4), two of which with microbleeds] and four matched control participants (**Table 3**). Of the individuals with SVD progression, all subjects with DWI+ lesions and monocyte purity >90% were selected to create a homogeneous subgroup. Participants with SVD progression were matched to those without SVD progression based on age, hypertension, and BMI.

Monocytes, with >90% purity in the isolated fraction, were stored in RNAprotect (Qiagen, Venlo, Netherlands) before processing for RNA sequencing using a standard RNA isolation protocol. In short, per 1 ml of RNAprotect, 200 μl of chloroform was added, mixed, incubated at room temperature for 5 min, and spun down for 15 min at 12,000 g at 4°C. The upper aqueous phase was transferred to an RNase-free Eppendorf tube, and an equal volume of 70% ethanol was added. After thorough mixing, the sample was loaded onto RNeasy mini columns (Qiagen), after which the manufacturer's protocol was followed. After the last manufacturer's step, 15 μl of RNase-free water was added to the columns, incubated for 5 min, and spun down. The RNA concentration was determined using the Qubit RNA HS assay (Qiagen), and the quality was determined using Nanodrop technology.

Library preparation was performed using the QuantSeq 3′ mRNA-Seq Library Prep Kit-FWD from Lexogen (Cat #015.96; Lexogen) according to the manufacturer's protocol. RNA input for all samples was normalized to 200 ng. All samples were processed in a single library preparation. The optimal cycle number for the endpoint PCR was determined on a 1:10 aliquot of the double-stranded cDNA libraries. Subsequent quality assessment, i.e. measuring the cDNA concentration using the Qubit dsDNA HS assay (Cat #Q32854; Thermo Fisher Scientific) and the average fragment size with the Agilent 2200 TapeStation (HS-D1000 ScreenTape, Cat #5067-5582; Agilent), enabled the determination of the molar concentration of individual libraries. Equimolar pooling of the libraries was set to 100 fmol, and after final dilution to 4 nM, libraries were sequenced on a NextSeq 500 instrument (Illumina; 1.4 pM loading concentration).

### Bioinformatics, Differential Gene Expression, and Pathway Analysis

Low-quality filtering and adapter trimming were performed using Trim Galore! V0.4.5 (Babraham Bioinformatics), a wrapper tool around the tools Cutadapt v1.18 and FastQC v0.11.5 (Babraham Bioinformatics). Reads were mapped to a human reference genome (GRCh38.95, Ensembl) with Star v2.6.0a (22), resulting in BAM files. BAM files were counted (number of reads mapped to a feature, e.g. a gene) with HTSeq [HTSeq-count tool v0.11.0 (23)] with default parameters using a complementary.gtf file, containing annotation for GRCh38.95 (Ensembl). MultiQC (quality control) was used to combine results and quality checks of all the samples (24). Total reads were between 8 and 11 million, of which 6–8.5 million were uniquely assigned reads; mean aligned reads was 80%.

Differential gene expression analysis was carried out with DESeq2 v1.22.0 in R v3.5.3 (25), with internal statistical and normalization method (i.e. correction for multiple testing with Benjamini–Hochberg) using a cutoff value of at least 5 counts (RPKM) per sample per gene. The average mRNA expression between two groups was analyzed: participants with incident DWI+ lesions were matched to controls without SVD progression while correcting for sex. Pathway analysis was performed with Reactome v.75 (26), selecting differentially regulated genes with an unadjusted *P*-value < 0.05 as input. A *P*-adjusted value < 0.05 with log2 fold change of > ±2 was considered significant. For pathway analysis, a false discovery rate (FDR)-corrected *P*-value < 0.05 was considered significant.

The data discussed in this publication have been deposited in NCBI's Gene Expression Omnibus (27) and are accessible through GEO.

### Statistics

Normal distribution of the data was checked with the Shapiro–Wilk test. When the *P*-value reached < 0.05, this assumption was violated, and non-parametric tests were used. In normally distributed data, the *P*-value of the independent samples *T*-test was used according to Levene's test for equality of variances. Normally distributed data are reported as mean ± SD and tested with independent samples *T*-test; categorical data are reported as mean with (number of participants) and tested with X^2^ test, and not normally distributed data are reported as median with interquartile ranges (IQRs) and tested with Mann–Whitney U-test.

Individuals with SVD progression were compared to subjects without incident lesions or WMH progression. In a separate analysis, subjects with incident lesions were compared to subjects without SVD progression. Circulating cell counts, inflammatory markers, and cytokine production capacity were log10-transformed and thereafter corrected with analysis of covariance (ANCOVA) for confounding demographics such as age, sex, and hypertension. A two-sided *P*-value < 0.05 was considered statistically significant.

## Results

### Participants Characteristics

**Figure 1:**
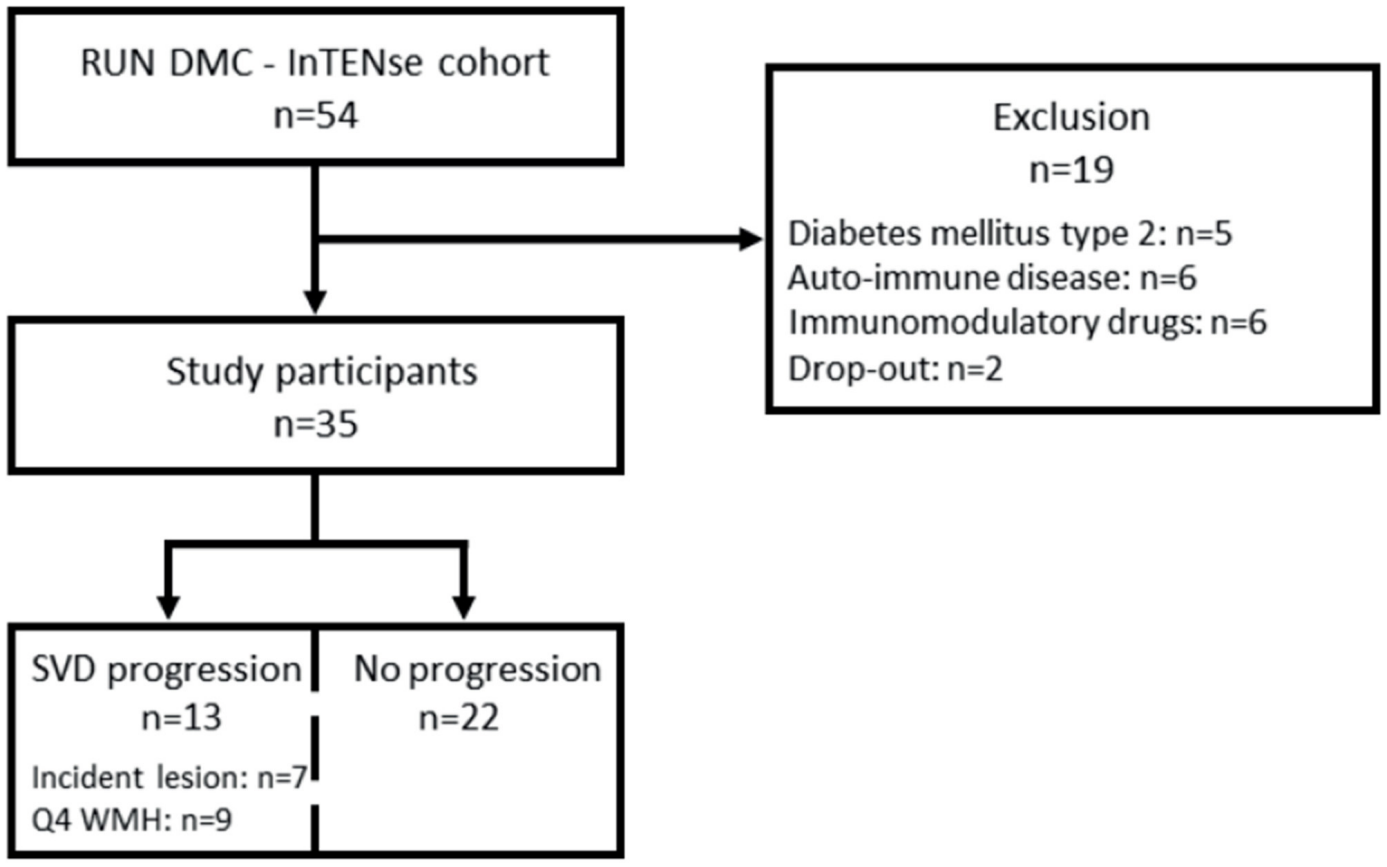
Flow diagram of included participants of the RUN DMC–InTENse cohort. Drop-out indicates premature stop before blood sampling. DM2, diabetes mellitus type 2; AID, autoimmune disease; drug use, chronic immunomodulatory drug use; 4Q WMH, highest quartile of WMH; WMH, white matter hyperintensity.

Thirty-five participants met the inclusion and exclusion criteria for this study [70 ± 6 years, 54% men, median follow-up time 39.3 (37.8–40.1) weeks] (see flow diagram in Figure 1). Over 10 months, the median WMH progression was 0.023 (0.002–0.078) ml per month (*n* = 35). In total, 13/35 individuals had SVD progression based on either incident lesions (*n* = 7) and/or the upper quartile of WMH progression (*n* = 9). Out of the seven individuals who developed incident lesions, five individuals had a total of 13 incident DWI+ lesions, three individuals had six incident microbleeds, and two individuals had five incident lacunes or small incident cavities. Two individuals had both DWI+ lesions and microbleeds and/or lacunes. Twenty-two individuals revealed neither incident lesions nor WMH progression in the highest quartile during 10 months of follow-up imaging (Figure 1).

Individuals with SVD progression were older (*p* < 0.01) and had a higher systolic (*p* < 0.01) as well as diastolic blood pressure (*p* < 0.05) compared to participants without SVD progression (Table 1). Similar results were obtained when analyses were restricted to participants with incident lesions. Therefore, all outcomes were corrected for the demographics age, hypertension, and sex, which are known modulators of the immune function (28).

**Table 1:** Participant characteristics.

**Demographics**	**Without SVD progression**	**SVD progression**	**Incident lesions**
Age, years	**68** **±** **4**	**73** **±** 7^**^	**77** **±** 7^**^
Sex, % men	50 (11)	62 (8)	71 (5)
BMI, kg/m^2^	25.0 ± 3.7	26.3 ± 4.2	**28.6** **±** 3.9^*^
SBP, mmHg	**136** **±** **15**	**155** **±** 23^**^	**162** **±** 23^**^
DBP, mmHg	**79** **±** **9**	**86** **±** 8^*^	**89** **±** 10^*^
Hypertension, %	82 (18)	92 (12)	100 (7)
Smoking, % active	5 (1)	8 (1)	0 (0)
Smoking, #packs/year	7.8 ± 10.8	11.1 ± 14.9	15.4 ± 17.9
Statin use, %	41 (9)	46 (6)	43 (3)
Acetylsalicylic acid use, %	32 (7)	62 (8)	71 (5)
Tchol, mmol/L	5.1 ± 1.1	4.9 ± 1.3	4.8 ± 1.6
HDL-C, mmol/L	1.6 ± 0.6	1.4 ± 0.4	**1.1** **±** 0.2^*^
LDL-C, mmol/L	2.9 ± 1.0	2.9 ± 1.2	3.1 ± 1.4
TG, mmol/L	1.4 ± 0.7	1.2 ± 0.4	1.3 ± 0.3
nHDL-C, mmol/L	3.6 ± 1.0	3.5 ± 1.3	3.7 ± 1.5
Non-fasting glucose, mmol/L	6.2 ± 1.4	6.0 ± 0.8	6.1 ± 0.9
Baseline WMH volume, ml	**2.25 (1.47–4.12)**	9.82 (5.54–22.2)^**^	6.88 (5.11–10.5)^*^
Baseline WMH, % of WM	**0.52 (0.373–1.03)**	2.20 (1.39–5.84)^**^	1.56 (1.31–3.19)^**^
WMH progression, ml/month	**0.02 (0.00–0.04)**	0.11 (0.01–0.20)^*^	0.01 (-0.04–0.20)
WMH progression, %0 of WM	**0.13 (0.03–0.27)**	0.62 (0.07–1.12)^*^	0.13 (-0.33–1.01)

*Participants without SVD progression (n = 22), SVD progression (n = 13), incident lesions (n = 7). Mean ± SD, mean (number), and median [IQR]*.

* *indicates P < 0.05*,

** *P < 0.01. BMI, body mass index; DBP, diastolic blood pressure; HDL-C, high-density lipoprotein cholesterol; IQR, interquartile range; LDL-C, low-density lipoprotein cholesterol; nHDL-C, non high-density lipoprotein cholesterol; SBP, systolic blood pressure; SVD, small vessel disease; TG, triglyceride; WMH, white matter hyperintensity. The bold values are statistically significant*.

### Higher Circulating E-Selectin in Participants With Small Vessel Disease Progression

Circulating E-selectin, a marker for endothelial dysfunction, was higher in participants with SVD progression (*p* < 0.05) (Table 2). Similar results were found when analyses were limited to participants with incident lesions. Cell counts in peripheral blood and subsets were comparable in participants with SVD progression to those without progression (Table 2).

**Table 2:** Circulating cells and inflammatory markers.

**Cell counts**	**Without SVD progression**	**SVD progression**	**Incident lesions**
WBC, 10^6^/ml	5.2 (4.7–6.7)	6.2 (5.1–7.3)	7.0 (4.8–7.7)
Neutrophils, 10^6^/ml	2.9 (2.3–3.6)	3.2 (2.5–4.5)	4.2 (2.6–5.0)
Lymphocytes, 10^6^/ml	1.8 (1.3–2.2)	2.0 (1.5–2.3)	1.9 (1.6–2.1)
Monocytes, 10^6^/ml	0.5 (0.4–0.7)	0.6 (0.4–0.7)	0.6 (0.3–0.7)
Monocytes, %	8.8 (7.5–11.0)	8.8 (7.4–10.5)	7.8 (6.7–9.8)
Classical monocytes, % gated	82.5 (78.0–88.0)^a^	80.2 (75.1–88.2)	79.6 (73.7–87.1)
Intermediate monocytes, % gated	7.9 (4.8–14.1)^a^	8.3 (4.5–11.9)	5.6 (2.5–11.7)
Nonclassical monocytes, % gated	7.0 (5.0–10.0)^a^	5.8 (4.5–12.5)	11.7 (5.4–13.2)
Circulating inflammatory markers			
hsCRP, pg/ml	1.4 (0.5–4.1)	1.1 (0.8–5.3)	1.3 (0.9–12.3)
hsIL-6, pg/ml	5.7 (3.3–9.6)	6.6 (4.0–11.4)^a^	5.1 (3.2–9.2)^a^
E-selectin, pg/ml	**13.9 (10.7–17.5)**	19.4 (16.8–24.3)^*^	20.0 (19.3–27.2)^*^
VCAM-1, pg/ml	392 (333–458)	410 (344–471)	424 (345–527)
MMP-2, pg/ml	898 (817–959)^a^	954 (863–1058)	968 (823–1,082)
CCL2, pg/ml^b^	31.2 (31.2–74.1)	36.8 (31.2–238)	43.5 (31.2–370)

*Participants without SVD progression (n = 22), SVD progression (n = 13), incident lesions (n = 7). Median (IQR). P-values are corrected for age, sex, and hypertension with ANCOVA*.

* *P < 0.05*.

a *Data are missing for one participant*.

b *Concentrations of CCL2 were often below the limit of detection of 31.2 pg/ml*.

*CRP, C-reactive protein; IL, interleukin; IQR, interquartile range; MMP, matrix metalloproteinase; SVD, small vessel disease; VCAM, vascular cell adhesion molecule; WBC, white blood cell. The bold values are statistically significant*.

### Increased Cytokine Production Capacity in Participants With Small Vessel Disease Progression

**Figure 2:**
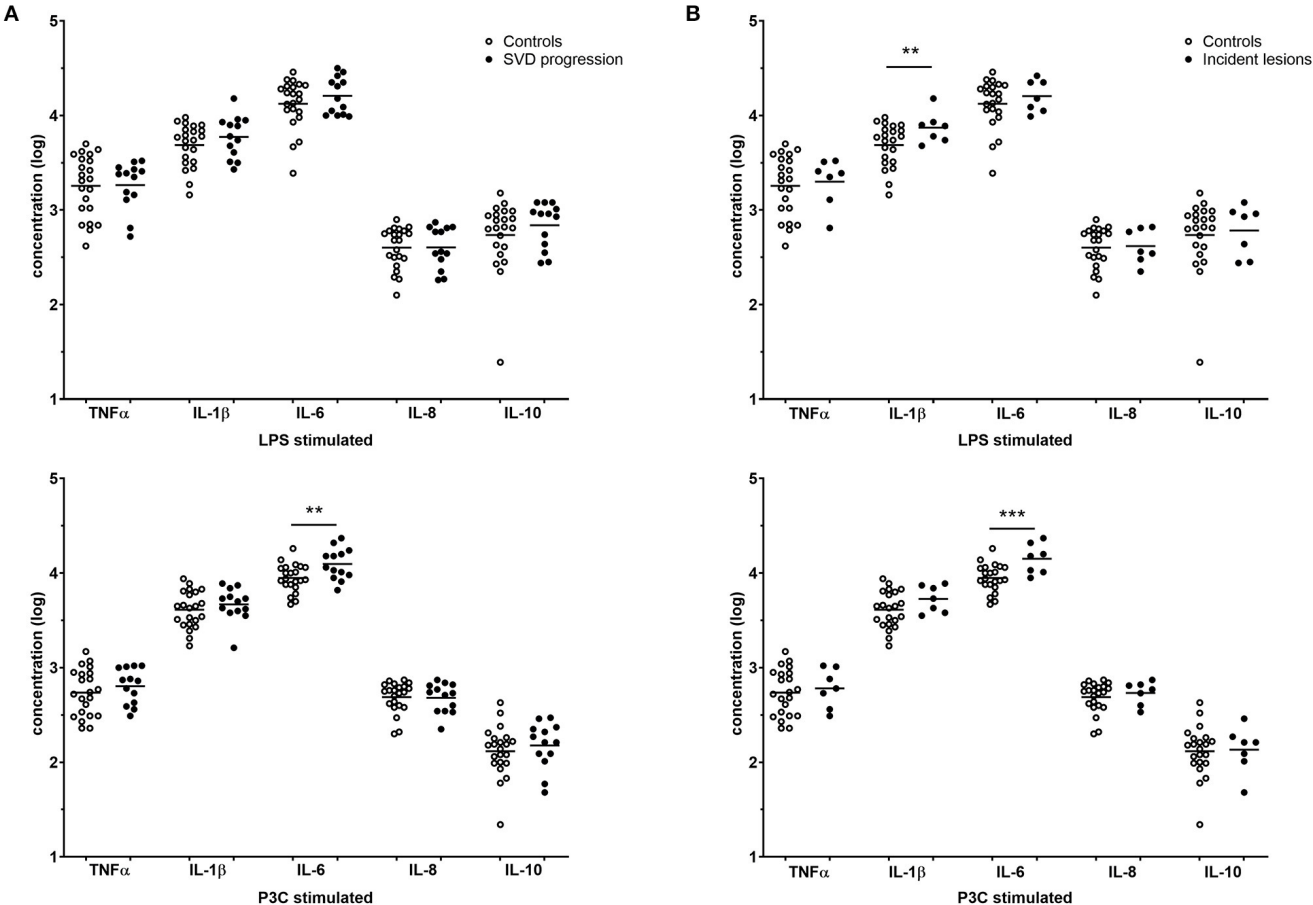
Cytokine production capacity. Cytokine responses of participants without SVD progression (white, *n* = 22), **(A)** participants with SVD progression (gray, *n* = 13) and **(B)** incident lesions (gray, *n* = 7) are reported as individual points with mean on log-transformed data. *P*-values are corrected for age, sex, and hypertension with ANCOVA. **P < 0.01, ***P = 0.001. SVD, small vessel disease.

The cytokine production capacity of isolated monocytes was significantly higher for IL-1β and IL-6 in individuals with SVD progression and was highly consistent for all incident lesions (Figures 2A,B). Individuals with SVD progression had higher IL-6 production after P3C stimulation compared to participants without SVD progression [median 11.4 (9.53–15.7) vs. 8.59 (7.61–11.4) ng/ml, *P* < 0.01] (Figure 2A). Likewise, in participants with incident lesions, IL-6 production after P3C stimulation was higher [15.2 (10.4–18.3) vs. 8.59 (7.61–11.4) ng/ml, *P* = 0.001] (Figure 2B). In addition, IL-1β production after LPS stimulation was higher in participants with incident lesions [7.76 (5.75–8.23) vs. 6.02 (3.35–7.66) ng/ml, *P* < 0.01]. Of note, in participants with DWI+ lesions and participants with microbleeds, similar results were obtained. No differences were observed in the production of the anti-inflammatory cytokine IL-10.

### Monocyte Transcriptome Analyses

In a subset of participants with SVD progression due to incident DWI+ lesions (*n* = 4) and matched participants without SVD progression (*n* = 4), we performed monocyte transcriptome analyses. The demographic and clinical characteristics of the two subgroups were comparable, except for sex (Table 3). Since the principal component analysis (PCA) plot also revealed sex-based clustering (Supplementary Figure 2), we controlled for sex in further differential gene expression analysis. This revealed a pro-inflammatory gene expression profile in participants with SVD progression due to incident DWI+ lesions.

**Table 3:** Participant characteristics of monocyte transcriptome analysis.

**Demographics**	**Without SVD progression**	**Incident lesions**
Age, years	71 ± 2	79 ± 9
Sex, % men	1 (25)	3 (75)
BMI, kg/m^2^	27.0 ± 4.2	28.2 ± 4.4
Hypertension	4 (100)	4 (100)
Smoking, % active	1 (25)	0 (0)
Baseline WMH volume, ml	**6.23 (1.41–10.54)**	**10.15 (6.28–23.27)**
Baseline WMH, % of WM	**1.54 (0.34–3.46)**	**2.69 (1.65–5.96)**
WMH progression, ml/month	**0.01 (0.00–0.05)**	**0.08 (0.00–0.20)**

*Participants with incident DWI+ lesions (n = 4) were matched to subjects without SVD progression (n = 4) for RNA sequencing. Mean ± SD, mean (number), and median (IQR). BMI, body mass index; DWI+, diffusion-weighted imaging-positive; IQR, interquartile range; SVD, small vessel disease; WMH, white matter hyperintensity. The bold values are statistically significant*.

**Figure 3:**
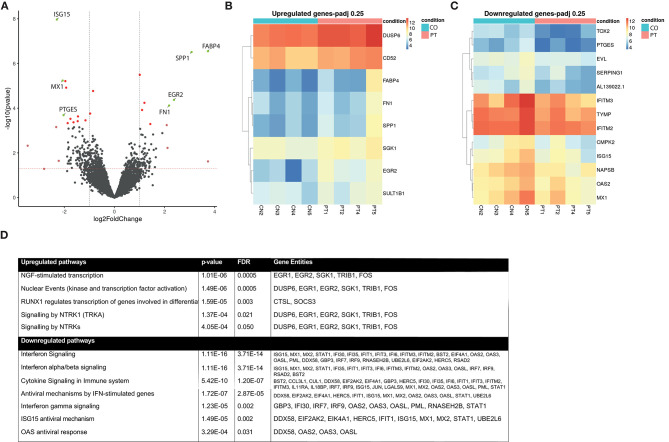
Monocyte transcriptome analysis. Differentially regulated gene expression between participants with SVD progression (*n* = 4) and without (*n* = 4). **(A)** Volcano plot of differentially expressed genes after sex stratification. Green dots indicate adjusted *P*-value < 0.25 and log Fold Change > ±2; Dark red dots, genes with *P*-adj < 0.25; light red dots, genes with logFC > ±2. **(B)** Heatmap of upregulated genes with *P*-adj < 0.25 corrected for sex. **(C)** Heatmap of downregulated genes with *P*-adj < 0.25 corrected for sex. Color coding is based on gene expression in raw counts, ranging from red (high expression value) to blue (low expression value). **(D)** Pathway analyses of significantly differentially expressed pathways after correction for sex, FDR < 0.05. Gene entities in each pathway were noted. FDR, false discovery rate; SVD, small vessel disease.

In the differential gene expression analysis, the two groups were compared in an explorative way using genes with a log fold change > ±2 and a liberate cutoff *P*-adjusted < 0.25. In this preliminary analysis, four genes were differentially upregulated and three were differentially downregulated (Figure 3A; Volcano plot): *FABP4, SPP1, EGR2, FN1* were upregulated, and *ISG15, MX1, PTGES* were downregulated. For a more detailed overview of the individual expression levels of the upregulated and downregulated genes with a *P*-adj < 0.25, we constructed heatmaps (Figures 3B,C).

Subsequently, we performed pathway analysis using all differentially regulated genes with an unadjusted *P*-value < 0.05 as input (supplementary Table 2). This revealed enrichment of several inflammation-related pathways and neuronal development and signaling pathways. These include the “*Nerve Growth Factor-stimulated transcription*” (FDR < 0.001), the “*Nuclear events*” (FDR < 0.001) pathway that is activated by neurotrophins, “*Signaling by Neurotrophic Receptor Tyrosine Kinase 1*” (FDR = 0.02) leading to proliferation of cell types and neuronal differentiation, and “*Signaling by Neurotrophic Tyrosine Kinase*” (FDR = 0.05) consisting of the receptor ligands for neurotrophins (Figure 3D). Downregulated pathways involved adaptive immune interferon (IFN) signaling, including IFN α, β, and γ signaling (FDR < 0.01), the “*ISG15* antiviral mechanism” pathway (FDR < 0.01), and “*Immune system*” pathway (FDR = 0.04) (Figure 3D). Similar results were obtained when the analyses were repeated without sex stratification (data not shown).

## Discussion

Our main finding is that a pro-inflammatory monocyte phenotype, characterized by an augmented cytokine production capacity, is associated with progression of SVD, as detected by serial high-frequency MRI scanning. In participants with SVD progression, circulating monocytes had a pro-inflammatory transcriptional signature, with significant upregulation of several inflammation-related pathways. These findings underscore our hypothesis that pro-inflammatory monocytes are closely involved in the development of SVD, uncovering innate immunity as novel potential pharmacological targets to prevent disease progression.

Given the overlap in pathophysiology and risk factors between atherosclerosis and SVD, we hypothesized that activation of circulating monocytes also contributes to SVD progression. The pathophysiology of SVD involves arteriolosclerosis, leading to arterial pathology of the smallest brain vessels, a process that is to a great extent comparable to large artery atherosclerotic disease. In addition, SVD shares cardiovascular risk factors with atherosclerotic disease, such as hypertension, dyslipidemia, and smoking. In atherosclerosis, we and others have shown that a pro-inflammatory phenotype of circulating monocytes, characterized by an augmented cytokine production capacity, is involved in the pathophysiology (29, 30).

Using serial MRI, we investigated the acute progression of SVD using imaging markers of SVD. We evaluated SVD progression in multiple ways: first by combining all SVD imaging markers (DWI+ lesions, microbleeds, lacunes, and WMH), then limited to incident lesions (DWI+ lesions, microbleeds, and lacunes), and finally each incident SVD imaging marker individually. We observed a higher cytokine production capacity of IL-6 and IL-1β in isolated monocytes after *ex vivo* stimulation in the individuals with SVD progression, which was consistent across subgroups of individuals with each incident SVD imaging marker evaluated separately. Previously, we described that the 9-year WMH progression preceding blood sampling correlated with the cytokine production capacity of monocytes, circulating inflammatory marker hsIL-6, and pro-inflammatory CD14^++^CD16^+^ monocytes in a retrospective cohort study of elderly individuals (14). The current high sequential imaging study enabled to increase the resolution with monthly imaging and to assess multiple imaging markers of SVD during acute progression of SVD.

To provide a deeper understanding of the pro-inflammatory monocyte phenotype, we explored the monocyte transcriptome with RNA sequencing in a small number of participants with and without SVD progression based on incident DWI+ lesions. We included participants with DWI+ lesions to create a homogeneous subgroup matched to subjects without SVD progression. The characteristics were comparable between subgroups, except for age, after which a sex-stratified analysis was performed. This explorative analysis revealed a pro-inflammatory gene expression profile with significant upregulation of several inflammation-related pathways together with a downregulation of adaptive immune IFN signaling pathways. Specifically, *EGR2* was upregulated, which encodes for a transcription factor that is essential for myelination of the nervous system, and defects result in peripheral neuropathies (31). Also, EGR2 and EGR3 are important for maintaining immune homeostasis (32). In innate immune cells, EGR2 expression is essential for naive or M2-like macrophages to respond to inflammatory stimuli (33). In amyloid-β plaque-associated microglia, a pro-inflammatory phenotype was found with upregulated *EGR2* and *SPP1* in a disease model for Alzheimer (34).

Another interesting observation is that two genes in the upregulated inflammation-related pathways encode for extracellular matrix components. Fibronectin (*FN*) and osteopontin-1 (*SPP1*) are principal components in cell matrix interactions, including within the blood–brain barrier. SPP1 also functions as an integrin, which mediates cellular adhesion, interaction, and is important in maintaining endothelial function. Additionally relevant in this context are two upregulated genes (FDR <0.25) involved in endothelial–leukocyte interaction. Both dual-specificity phosphatase 6 (*DUSP6*) and serum- and glucocorticoid-inducible kinase 1 (*SGK1*) orchestrate endothelial inflammation, increased expression of adhesion molecules in vascular tissue, and enhanced endothelial–leukocyte interaction mediating leukocyte recruitment (35, 36). More specifically, damage to the extracellular matrix is perpetuated by activated microglia or monocytes in response to hypoxia, eventually contributing to disruption of the blood–brain barrier (37). Chronic blood–brain barrier disruption enhances leakage of signaling mediators and facilitates the communication between circulating and tissue-resident immune cells and is thought to aggravate SVD (12). The fourth upregulated gene, fatty acid-binding protein 4 (*FABP4*) has been shown to be important in macrophage cholesterol trafficking, inducing foam cell formation and the development of atherosclerosis (38).

Remarkably, in conjunction with the upregulation of several inflammation-related pathways, we observed a downregulation of adaptive immune IFN signaling pathways in the monocyte transcriptome. Two out of three downregulated genes are involved in the interferon pathway. Ubiquitin-like protein (*ISG15*) and IFN-induced GTP-binding protein (*MX1*) are IFN-induced proteins that play central roles in the host antiviral response (39). Moreover, the third downregulated gene glutathione-dependent prostaglandin E synthase (*PTGES*) is also a major modulator of immune activation. This finding fits with the previously observed negative association between the *ex vivo* IFN-gamma production capacity and SVD progression (14). We previously described that this might point to a counter-regulatory mechanism between innate and adaptive immunity.

Pathway analysis revealed several pathways involved in neuronal development and differentiation and signaling by neurotrophins, which are pivotal proteins in neuronal survival, growth, differentiation and during development. There are several lines of evidence indicating that neurotrophins play important roles in the pathophysiology of neurodegenerative and psychiatric disorders (40). It is intriguing that circulating monocytes of individuals with SVD show a higher expression of genes in pathways involved in neuronal development. It is relevant to gain a better insight in the influence of circulating monocytes through neurotrophins on neurons in the brain.

To our knowledge, we are the first to study the monocyte transcriptome in individuals with SVD. Previously, whole-blood gene expression in the Framingham Heart cohort (*n* = 3,248) showed that WMH was associated with genes of inflammation-related pathways (41). The heightened expression of inflammatory genes and pathways of monocytes in our studied cohort fits with their increased immunologic activity. Moreover, it reveals possible underlying mechanisms, with the *EGR2* gene required in myelination, extracellular matrix components that mediate blood–brain barrier integrity, *DUSP6* and *SGK1* that trigger endothelial leukocyte recruitment, and neurotrophins that are essential for neurons—all processes involved in the complex pathophysiology of SVD.

With the current findings, it is tempting to speculate about the mechanisms responsible for this pro-inflammatory monocyte phenotype. Monocyte activation can be due to genetic variation that predisposes to hyperresponsive monocytes (42) or due to stimulation of monocytes by circulating stimuli, such as elevated levels of lipoproteins. Finally, trained immunity might contribute to persistent monocyte hyperresponsiveness (43). Dyslipidemia, either caused by a Western-type diet or due to familial hypercholesterolemia, induces hyperresponsiveness of circulating monocytes, which persists despite normalization of plasma cholesterol levels (44, 45). This is, at least in part, mediated by metabolic and epigenetic reprogramming of these monocytes. Trained immunity has recently been suggested in the context of Alzheimer's disease; brain-resident microglia can develop immunological memory after repeated administration of LPS in the circulation in animal models (46), inducing a persistent elevated cytokine production mediated by epigenetic reprogramming. The pro-inflammatory responses by the trained microglia accelerated disease progression in an Alzheimer's disease model. Currently, it remains a question for future investigations whether the pro-inflammatory monocyte phenotype is due to differences in the genetic code, due to persistent stimulation of monocytes with circulating factors, or due to epigenetic reprogramming in the context of trained immunity.

A potential limitation of this study is the small sample size due to its complex and intensive design. Although in the current study design the monocyte phenotyping preceded the progression of SVD, we cannot draw conclusions on causality, since it is likely that SVD progression was also present in participants with incident lesions before inclusion in the study.

In conclusion, the pro-inflammatory monocyte phenotype and transcriptome, characterized by an increased cytokine production capacity and augmentation of inflammatory pathways, are related to the progression of SVD in elderly individuals. Future studies are needed to evaluate the mechanisms responsible for monocyte activation, including the potential role for trained immunity, and to provide the causality whether these activated monocytes initiate SVD progression.

## Data Availability Statement

The datasets presented in this study can be found in online repositories. The names of the repository/repositories and accession number(s) can be found below: https://www.ncbi.nlm.nih.gov/geo/, GSE162790.

## Ethics Statement

The studies involving human participants were reviewed and approved by Institutional Review Board Arnhem/Nijmegen, the Netherlands (NL53939.091.15). The patients/participants provided their written informed consent to participate in this study.

## Author Contributions

AT, KW, AMT, MD, and F-EL designed and performed the RUN DMC–InTENse study. MPN, NR, MGN, and LJ designed the current study. MPN performed the experiments and data analysis and wrote the manuscript. CK, SK, SB, CH, and AH accomplished RNA sequencing or analysis. All authors contributed to the manuscript and approved the submitted version.

## Conflict of Interest

The authors declare that the research was conducted in the absence of any commercial or financial relationships that could be construed as a potential conflict of interest.

## Abbreviations

SVD: cerebral small vessel disease
WMH: white matter hyperintensity
MRI: magnetic resonance imaging
IL-6: interleukin-6
CRP: C-reactive protein
RUN DMC–InTENse: Radboud University Nijmegen Diffusion tensor and Magnetic resonance imaging Cohort–Investigating The origin and EvolutioN of cerebral small vessel disease
DWI: diffusion-weighted imaging
BMI: body mass index
IMT: intima media thickness
Tchol: total cholesterol
HDL-C: high-density lipoprotein cholesterol
TG: triglyceride
LDL-C: low-density lipoprotein cholesterol
RPMI: Roswell Park Memorial Institute
LPS: lipopolysaccharide, toll-like receptor 4 agonist
P3C: Pam3CysK4, toll-like receptor 2 agonist
TNFα: tumor necrosis factor alpha
EGR2: endothelial growth response 2
RUNX3: Runt-related transcription factor 3
TGF-β: tumor growth factor beta
SPP1: osteopontin-1.
