# Supplementary material for: Pro-inflammatory Monocyte Phenotype During Acute Progression of Cerebral Small Vessel Disease
Source: Front Cardiovasc Med. 2021 May 13;8:639361. doi: 10.3389/fcvm.2021.639361 (PMC8155247; doi:10.3389/fcvm.2021.639361)
Supplement: Supplementary file 1 [file Data_Sheet_1.docx]

**Supplementary material**

**Title: Pro-inflammatory monocyte phenotype during acute progression of cerebral small vessel disease**

**Supplemental Methods**

RNA isolation, sequencing and analysis

Low quality filtering and adapter trimming was performed using Trim Galore!, V0.4.5 (Babraham Bioinformatics), a wrapper tool around the tools Cutadapt v1.18 and FastQC v0.11.5 (Babraham Bioinformatics). Reads were mapped to a human reference genome (GRCh38.95, Ensembl) with Star v2.6.0a (1) using a pre-built genome index with fasta and gtf from Ensembl while using the parameter "--sjdbOverhang 100", with STAR (v 2.6.0a). For the alignment the non-default parameter "--outSAMtype BAM Unsorted" was used. BAM files were counted (number of reads mapped to a feature, e.g. a gene) with HTSeq (HTSeq-count tool v0.11.0 (2)) with default parameters using a complementary .gtf file, containing annotation for GRCh38.95 (Ensembl). MultiQC (quality control) was used to combine results and quality checks of all the samples (3).

Differential gene expression analysis was carried out with DESeq2 v1.22.0 in R v3.5.3 using the packages DESeq2 (v 1.22.2), data.table (v 1.12.8), ggplot2 (v 3.3.2), edgeR (v 3.24.3), ggrepel (v 0.8.1), pheatmap (v 1.0.12), RColorBrewer (v 1.1-2) and tidyr (v 1.1.2) (4). Internal statistical and normalization method (*i.e.* correction for multiple testing with Benjamini–Hochberg) using a cut-off value of at least 5 counts per sample per gene. Pathways analysis was performed with Reactome v.75 (5) selecting differentially regulated genes with a *P*-value <0.05 as input.

**Supplemental Tables**

Table I. Products details of cytokines and chemokines measured with ELISA.

|  | Item | Manufacturer | Category number |
| --- | --- | --- | --- |
| 24h stimulation | Human TNFα duoset | R&D | DY210 |
|  | Human IL-1β duoset | R&D | DY201 |
|  | Human IL-6 duoset | R&D | DY206 |
|  | Human Prelipair IL-8 | Sanquin | M9318 |
|  | Human Prelipair IL-10 | Sanquin | M9310 |
| Plasma | Human hs-CRP quantikine | R&D | DCRP00 |
|  | Human hs-IL-6 quantikine | R&D | HS600C |
|  | Human VCAM-1 duoset | R&D | DY809 |
|  | Human E-selectin duoset | R&D | DY724 |
|  | Human MMP-2 duoset | R&D | DY902 |
|  | Human CCL2 duoset | R&D | DY279 |

Table II. Input of differently regulated genes for pathway analysis

| Upregulated genes | | | | Downregulated genes | | | | |
| --- | --- | --- | --- | --- | --- | --- | --- | --- |
| FABP4 | RIOK2 | FOS | BCL9L | ISG15 | RSAD2 | SIGLEC1 | ADAM15 |  |
| SPP1 | IKBIP | ARNTL | LARP1B | MX1 | AL035071.1 | PES1 | MARCKS |  |
| DUSP6 | ZNF808 | AC010343.1 | DHX16 | IFITM3 | P2RY2 | SH3BP2 | TMEM160 |  |
| EGR2 | CCDC126 | TMTC2 | RGS18 | AL139022.1 | GTPBP1 | ADCY9 | ZNF789 |  |
| SGK1 | PCNX4 | GPATCH11 | CR1 | IFITM2 | AC018628.1 | IL18BP | ITPK1-AS1 |  |
| FN1 | C14orf119 | NDUFS8 | SOCS6 | EVL | GABBR1 | RNASEK | RNASEH2B |  |
| CD52 | F11R | ZNF614 | SGPL1 | PTGES | HELZ2 | LY6E | PATL2 |  |
| SULT1B1 | FAM50B | BCL9L | TLR4 | OAS2 | AP2A1 | SCNM1 | AC135178.3 |  |
| HLCS | UTP15 | LARP1B | ACP5 | SERPING1 | AC008764.10 | USP19 | APBB3 |  |
| NCAPD2 | ZNF143 | DHX16 | DNAJC3 | TYMP | AC008982.2 | AP000695.3 | HCST |  |
| FABP5 | KCNJ15 | RGS18 | MRPL22 | NAPSB | AC007365.1 | POLG2 | RUNX3 |  |
| HEMGN | TMEM38B | CR1 | ABCD3 | TOX2 | TBXAS1 | FADS3 | SPNS1 |  |
| RGS2 | GIHCG | SOCS6 | RFC5 | CMPK2 | RAC1P2 | APOBEC3A | NEDD1 |  |
| BTG2 | TMEM168 | SGPL1 | CXCR2 | U62317.2 | PARP12 | TMCC1 | VPS9D1 |  |
| TSPYL4 | MGME1 | TLR4 | LRRC37A16P | AC008533.1 | C7orf50 | KLHDC10 | DHRS4L2 |  |
| KIAA1551 | TRIB1 | ACP5 | DUS4L | GBP3 | ZEB2-AS1 | RBM4 | DNPEP |  |
| CYBRD1 | PBDC1 | DNAJC3 | LRRFIP2 | SMPD3 | RRN3P2 | PTK6 | DDX60 |  |
| TRIP11 | SCD | MRPL22 |  | IFIT1 | USP21 | CUL1 | TMEM175 |  |
| ZNF200 | TMEM242 | ABCD3 |  | AC073869.1 | ZNF408 | BST2 | NME9 |  |
| ZSCAN29 | LIPN | RFC5 |  | EIF4A1 | TSPAN4 | C5orf56 | RPL36A |  |
| DYNLT3 | CDKL5 | CXCR2 |  | PARP14 | AL731571.1 | GON4L | RWDD2B |  |
| ANKRD28 | RSU1 | LRRC37A16P |  | EIF2AK2 | PPAN | RF00019 | ZNF532 |  |
| EGR1 | LPAR1 | DUS4L |  | LENG8 | IRF7 | CLEC12B | NFATC2IP |  |
| FMC1 | TRIM13 | LRRFIP2 |  | TARBP1 | MAN2B1 | CYP2S1 | LGALS2 |  |
| NAGS | AC118549.1 | PSD3 |  | FXYD6 | ZBP1 | MB21D2 | CAPN10 |  |
| CENPW | KIAA1328 | INTS2 |  | PRMT9 | FAM160B2 | UROS | TARBP2 |  |
| CREB5 | MCCC2 | PPT2 |  | BLVRA | TMEM91 | MED10 | RF00019 |  |
| RHOBTB1 | ACSM3 | S100A12 |  | PML | LY6G5C | AP000347.1 | ULK4 |  |
| ENC1 | COQ10B | TP53I11 |  | ACAP3 | OAS3 | KCNMB1 | RNU6-1016P |  |
| TMEM41B | PLBD1 | PHF10 |  | MX2 | C12orf43 | MTMR12 | CAPNS1 |  |
| TCTN3 | BCAT1 | SNAP47 |  | JUN | STAT1 | OXCT1 | MASTL |  |
| AC093673.1 | MARC1 | SGMS1 |  | SPATA20 | PNPT1 | AC003681.1 | AC012368.1 |  |
| NDUFA12 | NFE2L3 | TOP1 |  | PLEKHM1P1 | FCGR2B | NSUN5P1 | OSBPL5 |  |
| NRG1 | SOWAHC | PALLD |  | RNF213 | ENGASE | NDRG2 | TNFAIP3 |  |
| CTSL | S100A12 | ARHGEF12 |  | RNU4ATAC | SNRPN | EPSTI1 | DUSP18 |  |
| ZBTB38 | TP53I11 | HTRA1 |  | FMNL1 | SNHG20 | IFI6 | OXNAD1 |  |
| TMEM206 | PHF10 | ERLEC1 |  | L3MBTL2 | CD1E | FAHD2A | TMC8 |  |
| TLR1 | SNAP47 | KCNE3 |  | SUMF2 | TRABD | SCAMP5 | PLEKHG3 |  |
| ASGR1 | SGMS1 | FPR2 |  | IFI44L | ALG3 | IFIT3 | UPK3A |  |
| THBD | TOP1 | ATP2A2 |  | LGALS9 | LGALS3BP | MED17 | AP001107.1 |  |
| DUSP2 | PALLD | GPRIN3 |  | PDLIM7 | DTX3L | TRAF5 | KMT2E-AS1 |  |
| DIPK2A | ARHGEF12 | SLC16A6 |  | NAA40 | HSF2 | TCEA2 | CPEB2 |  |
| SMIM13 | HTRA1 | NRBF2 |  | AC025171.1 | AD001527.1 | ZCCHC2 | CCDC57 |  |
| FADS2 | ERLEC1 | YWHAE |  | TMEM220 | GMIP | UBE2L6 | HERC5 |  |
| METTL9 | KCNE3 | SOCS3 |  | CCL3L1 | SIRT7 | SH3PXD2B | PPP3CB-AS1 |  |
| TENT2 | FPR2 | CRTAM |  | LILRA6 | CHMP4A | ZNF296 | ZBTB42 |  |
| ERI1 | ATP2A2 | CEP19 |  | SNORD13E | NUDT4 | AP002762.1 | AC109460.1 |  |
| FOLR3 | GPRIN3 | C17orf51 |  | AC010997.6 | ZFAND2A | IFI30 | MRPS2 |  |
| ZNF232 | SLC16A6 | EREG |  | SHISA5 | ADAT2 | BCL7A | SH3GLB2 |  |
| SLC23A2 | NRBF2 | CBX7 |  | DDX58 | JUP | SAMD4A | CCT6B |  |
| RPH3A | YWHAE | ARRDC4 |  | IRF9 | OASL | ANKRD52 | PARP9 |  |
| FEM1C | SOCS3 | IFT20 |  | NEAT1 | IL11RA | TPRG1L | CUL9 |  |
| ZUP1 | CRTAM | IL7R |  | ODF3B | SELL | AC004241.3 | EPM2AIP1 |  |
| CEP170 | CEP19 | F8A1 |  | LILRA4 | ACBD6 | ZNF335 | CST3 |  |
| STK38L | C17orf51 | ANPEP |  | C19orf66 | IFI35 | SMARCE1 | SPATS2L |  |
| ZNF503 | EREG | ICA1 |  | CMTR1 | OSCAR | ATF5 | LINC00173 |  |
| ITGAV | CBX7 | THAP12 |  | VASH1 | CENPF | AL035587.1 | AP2S1 |  |
| ZNF354A | ARRDC4 | QARS |  | EIF5 | RNU4-2 | OAZ2 | MVB12B |  |
| MOGS | IFT20 | FOS |  | DNASE1L1 | FANCF | AC073957.3 | CHST13 |  |
| DSE | IL7R | ARNTL |  | ID2 | IER5 | CD1C | GPSM3 |  |
| PTGER4 | F8A1 | AC010343.1 |  | UCP2 | IFI44 | SRGAP2C | PLEKHM2 |  |
| GTPBP10 | ANPEP | TMTC2 |  | C1orf56 | AC069549.1 | DCAKD | SEMA4A |  |
| AC005332.6 | ICA1 | GPATCH11 |  | VSTM1 | BHLHE40 | RBM3 |  |  |
| TMEM176B | THAP12 | NDUFS8 |  | AC109326.1 | AC099343.3 | C1orf115 |  |  |
| NRIP3 | QARS | ZNF614 |  | CELF2-AS1 | WDR27 | SH3TC1 |  |  |

Differentially regulates genes with an unadjusted p-value<0.05 were included in Reactome pathway analysis.

**Supplemental Figures and Figure legends**

Figure I. Gating strategy.


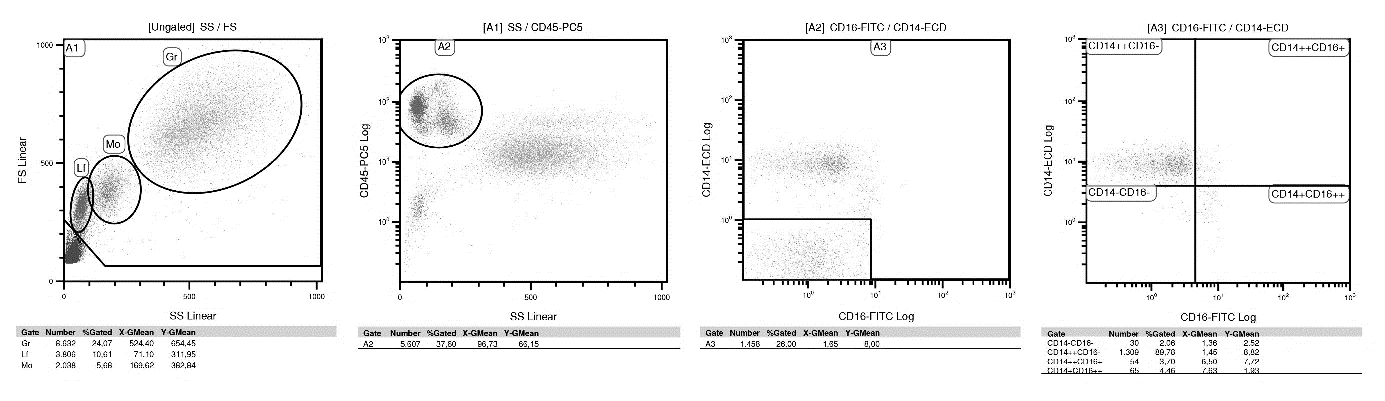
Monocytes were gated in SSC/CD45+ plot, identifying monocytes as CD45+ cells with monocyte scatter properties. Exclusion of lymphocytes and natural killer cells was performed by excluding CD14/CD16 negative cells. Percentages of monocyte subsets (CD14^++^CD16^−^, CD14^++^CD16^+^, and CD14^+^CD16^++^) were identified in the CD14/CD16 plot.


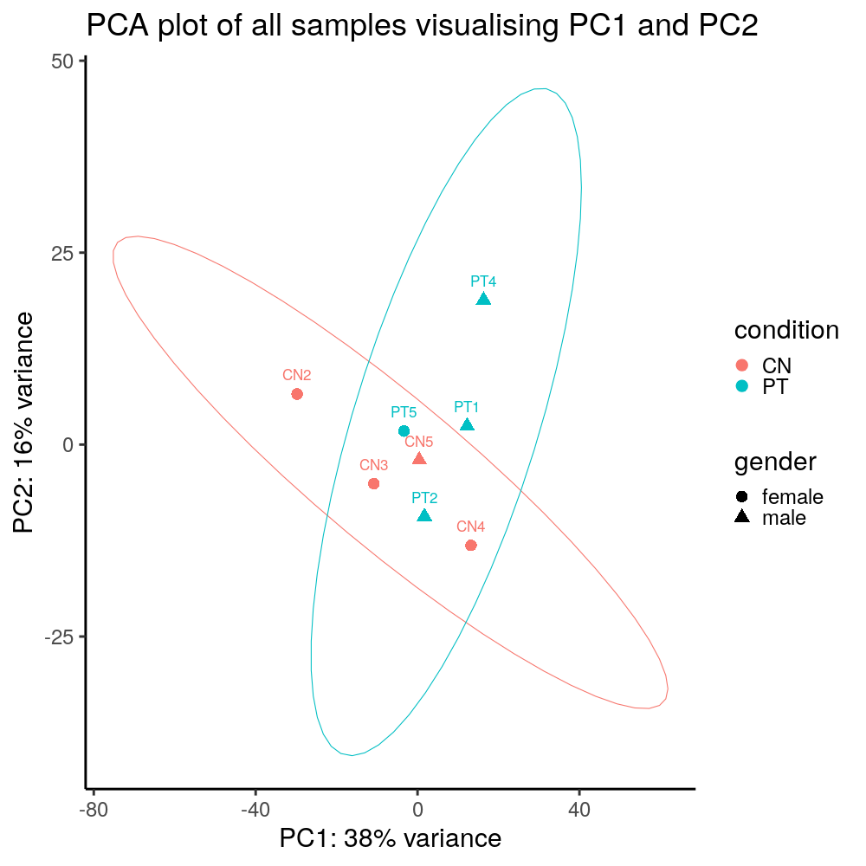
 Figure II. PCA plot of monocyte transcriptome analyses.

PCA plot of differentially regulated gene expression between participants with SVD progression based on DWI+ lesions (n=4, blue dots) and without (n=4, red dots). Circles indicate women, triangle: men.

**References**

1. Dobin A, Davis CA, Schlesinger F, Drenkow J, Zaleski C, Jha S, et al. STAR: ultrafast universal RNA-seq aligner. Bioinformatics. 2013;29(1):15-21.

2. Anders S, Pyl PT, Huber W. HTSeq--a Python framework to work with high-throughput sequencing data. Bioinformatics. 2015;31(2):166-9.

3. Ewels P, Magnusson M, Lundin S, Kaller M. MultiQC: summarize analysis results for multiple tools and samples in a single report. Bioinformatics. 2016;32(19):3047-8.

4. Love MI, Huber W, Anders S. Moderated estimation of fold change and dispersion for RNA-seq data with DESeq2. Genome Biol. 2014;15(12):550.

5. Fabregat A, Sidiropoulos K, Viteri G, Forner O, Marin-Garcia P, Arnau V, et al. Reactome pathway analysis: a high-performance in-memory approach. BMC Bioinformatics. 2017;18(1):142.
